# A Case Report of Rapid Vision Improvement in a Patient With Optic Nerve Sheath Meningioma Treated Using ZAP-X Radiosurgery

**DOI:** 10.7759/cureus.98108

**Published:** 2025-11-29

**Authors:** Yang Bai, Longsheng Pan, Jingmin Bai, Jinyuan Wang, Zijing Zeng, Yunmo Liu

**Affiliations:** 1 Department of Neurosurgery, People's Liberation Army (PLA) General Hospital, Beijing, CHN; 2 Department of Radiotherapy, People's Liberation Army (PLA) General Hospital, Beijing, CHN; 3 Department of Radiation Oncology, People's Liberation Army (PLA) General Hospital, Beijing, CHN

**Keywords:** accurate dosing, brain stereotactic radiosurgery, optic nerve sheath meningioma (onsm), vision therapy, zap-x radiosurgical system

## Abstract

Optic nerve sheath meningioma (ONSM) is a rare benign tumor of the central nervous system. Although lesions in this region typically grow slowly, their proximity to the optic nerve directly impacts the frontal visual pathway and may lead to severe vision problems. The mainstream treatment for ONSM has shifted from traditional surgery to stereotactic radiosurgery (SRS). Among these, ZAP-X (ZAP Surgical Systems, Inc., San Carlos, California), the latest generation of SRS systems, offers a novel approach to clinical treatment of ONSM with its noncoplanar rotational focus technology, six-dimensional dynamic tracking, and submillimeter (<0.4 mm) treatment precision. This system avoids traditional head-pin injuries and reduces radiation trauma to surrounding healthy tissues, while providing short-duration, high-efficiency treatment with integrated intelligence, real-time imaging verification, and adaptive planning features.

We report the case of a 61-year-old male patient who had worn glasses for over 20 years and presented with progressive vision deterioration in the right eye for more than two years. Conventional vision correction was ineffective, prompting him to seek medical attention. Cranial and orbital magnetic resonance imaging (MRI) revealed a localized lesion in the intraorbital segment of the right optic nerve, showing the "tram-track sign." Demyelination tests indicated negative results for serum autoantibodies against aquaporin-4 and myelin oligodendrocyte glycoprotein (MOG). Pretreatment examinations revealed corrected visual acuity: 0.5 (left eye), 0.1 (right eye); normal appearance of the left eyeball, while the right eyeball was externally deviated by 45°; both pupils were round and equal in diameter (0.3 mm), with a slightly sluggish light reflex in the right eye and positive relative afferent pupillary defect sign (+). Intraocular pressure was 15 mmHg (left) and 14 mmHg (right). Fundus examination showed a white optic disc in the right eye, with significantly tortuous and dilated retinal veins forming a "spiral" pattern, while the contralateral fundus was unremarkable. Based on these findings, the patient was ultimately diagnosed with primary ONSM in the right eye. Due to the patient's high expectations for vision preservation, ZAP-X SRS was chosen as the treatment modality. Postoperative follow-up over 1.5 years showed good visual recovery, with stable clinical and imaging findings.

As a globally advanced radiotherapy modality, this case suggests that the ZAP-X system may provide a safe and effective treatment option for ONSM. The patient experienced significant visual field recovery immediately after the initial fractionated treatment, with long-term stability in visual acuity, prompting us to report this case.

## Introduction

Optic nerve sheath meningioma (ONSM) is a benign tumor originating from arachnoid cap cells surrounding the optic nerve sheath, accounting for 1%-2% of all meningiomas and 10% of orbital tumors [1]. Pathologically, it is classified mostly as WHO Grade 1, and its lesion structure grows slowly. However, its anatomical location near the optic nerve and anterior clinoid process can cause vision impairment due to optic nerve compression [2]. Clinically, it often manifests as painless progressive vision loss (main symptom), visual field defects, or color vision abnormalities. In severe cases, it may lead to blindness. The tumor's mass effect can increase orbital pressure, causing exophthalmos and incomplete eyelid closure symptoms.

Diagnosing ONSM is challenging and usually requires a combination of imaging and ophthalmological examinations. Common imaging modalities include cranial and orbital MRI (enhanced scans) and magnetic resonance angiography for excluding vascular lesions or assessing tumor vascular supply. Ophthalmological examinations are typically more complex, involving visual acuity and visual field assessments. Fundus examinations are particularly indicative of optic disc edema and atrophic changes. For instance, optic nerve sheath veins may show obstruction due to tumor mass effect, resulting in "serpentine" or "spiral" tortuosity. Advanced stages often exhibit optic disc edema followed by secondary optic nerve atrophy. Additionally, abnormal collateral vessels on the optic disc surface or margins may cause diplopia or ocular movement disorders.

Traditional treatment methods include observation and surgical intervention, but neither offers optimal vision preservation. Conventional decompression surgery has poor therapeutic outcomes and often results in irreversible damage to vision function [3,4].

In recent years, stereotactic radiosurgery (SRS) has gradually become the preferred treatment modality for ONSM [5]. Among these, the ZAP-X system, as the latest noninvasive radiosurgical platform, employs non-coplanar arc-shaped intensity-modulated radiation to achieve millimeter-level targeting precision and dynamic conformal technology, bringing breakthrough advancements in ONSM treatment. Its self-shielding structure and real-time 3D tracking system also address the conventional SRS limitations of respiratory-induced displacement issues [6]. This provides a novel therapeutic approach for intracranial tumors, especially those requiring organ function preservation in intracranial space-occupying lesions.

## Case presentation

This case involves a 61-year-old male patient who presented to the ophthalmology outpatient clinic of the Chinese PLA General Hospital with a chief complaint of progressive vision loss in the right eye for over two years. The patient’s medical history revealed that since October 2019, he had noticed darkened vision and gradual vision deterioration in the right eye without any apparent cause. Due to the absence of other discomforting symptoms, the patient initially ignored the condition. In September 2020, he observed that vision correction did not improve his eyesight.

In March 2021, the patient sought consultation at our neurosurgery department. Imaging studies, including cranial and orbital MRI, revealed a localized lesion in the intraorbital segment of the right optic nerve, with iso-T1 and slightly hyper-T2 signals, and T1 enhancement showing the "tram-track sign." Ophthalmological examinations indicated left eye visual acuity of 0.5 and right eye visual acuity of 0.1; the left eyeball was normal, while the right eyeball was externally deviated by 45°. Both pupils were equal in size, with the left eye showing no abnormalities and the right pupil exhibiting relative afferent pupillary defect sign (+) and sluggish light reflex. Fundus examination revealed no abnormalities in the left eye, while the right optic disc was white with clear boundaries, the C/D ratio was approximately 0.3, and the retinal vessels were significantly tortuous and dilated in a "spiral" pattern, along with optic nerve atrophy in the right eye. Demyelination tests (serum aquaporin-4 and MOG) were negative, ruling out optic nerve demyelination. Based on the patient’s clinical findings and imaging results, a multidisciplinary team (MDT) led by neurosurgeons and comprising ophthalmologists, radiologists, and radiotherapy technologists collectively diagnosed the patient with "primary ONSM in the right eye" [7].

The patient underwent SRS using the ZAP-X system (ZAP Surgical Systems, Inc., San Carlos, California) from April 12 to April 17, 2021, with a total prescription dose of 23 Gy-5F (4.6 Gy per session). Pretreatment imaging included 1-mm-thick CT and MRI scans for localization, followed by rigid image fusion of the MRI Bravo enhanced sequence with CT images using MIM-Maestro 6.5.4 software (MIM Software, Inc., Cleveland, Ohio), which facilitated target area delineation for the treatment field.

**Figure 1:**
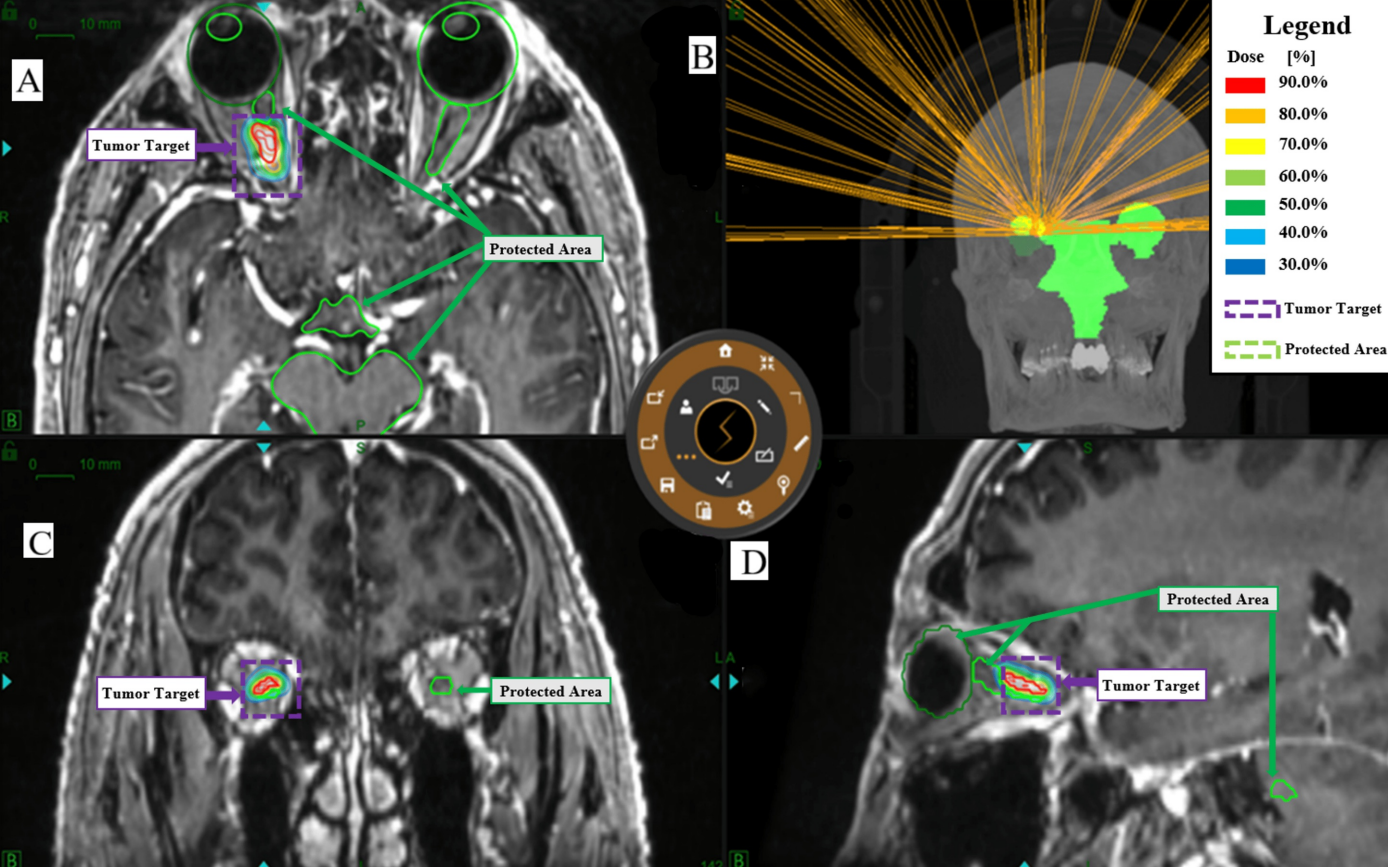
The tumor target area and nearby critical tissues during the patient's treatment planning. (A) Axial, (C) coronal, and (D) sagittal views of the target volume delineation. (B) The radiation beam path during treatment. The marked tumor region represents the irradiation zone for the optic nerve sheath meningioma within the ZAP-X stereotactic radiosurgical system. The different colored zones indicate the steep dose gradient in the surrounding tissues. The red border marks where the radiation dose to adjacent tissues has decreased to 90% of the total dose, while the blue border indicates a reduction to 30%, demonstrating effective protection of healthy structures nearby. The outlined protective areas represent critical neighboring structures; their contouring helps optimize treatment planning by minimizing radiation exposure to these regions and reducing the risk of side effects

The VIDAR™ (VIDAR Systems Corporation, Herndon, Virginia) eye-tracking system integrated into the ZAP-X platform enables monitoring and intervention of core eye movements. It primarily tracks the pupillary margin of the patient’s eye and its dynamic changes. Prior to treatment initiation, an eye-tracking calibration procedure will be conducted, with a threshold value set at 0.3 mm. This threshold defines the maximum permissible range of eye movement during treatment, ensuring treatment safety. Figure 1 shows the ZAP-X radiation therapy plan for this patient.

**Figure 2:**
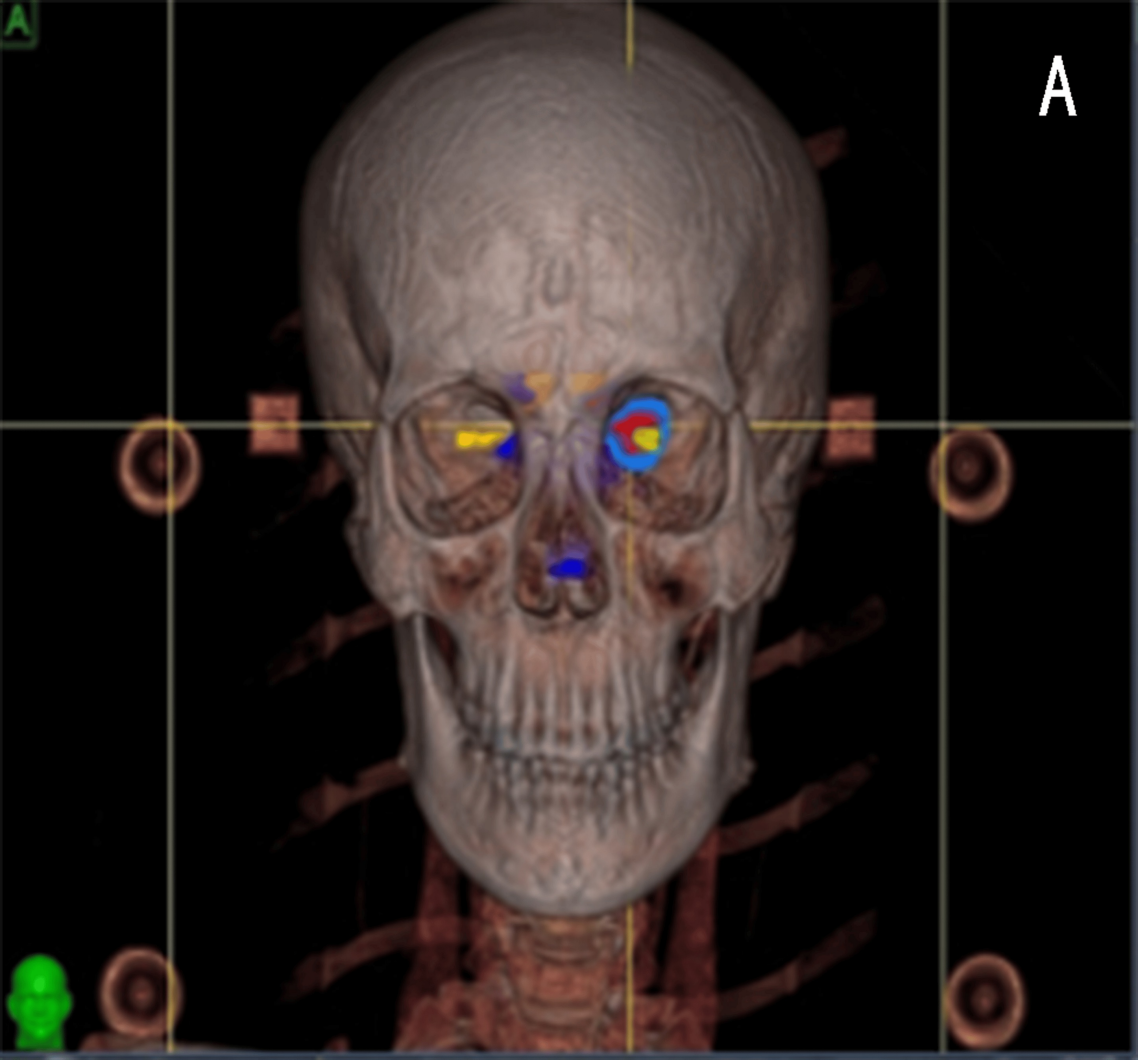
Delineation diagram of the optic nerve protection zone

Figure 2 presents a radiotherapy design diagram showing the delineation of the radiation target area for the patient, followed by a secondary delineation of the optic nerve-sparing area.

**Figure 3:**
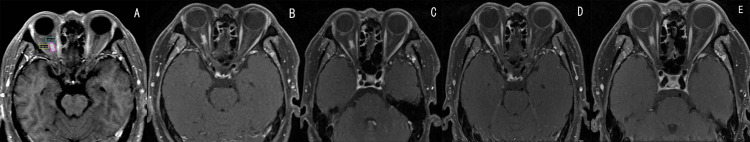
MRI scan images (A) Preoperative MRI scan images. (B) MRI scan image one month after ZAP-X treatment. (C) MRI scan image three months after ZAP-X treatment. (D) MRI scan image six months after ZAP-X treatment. (E) MRI scan image 18 months after ZAP-X treatment MRI: magnetic resonance imaging

Figure 3 presents MRI scan images during the follow-up period. Due to the small tumor size, MRI scans did not reveal significant changes in tumor volume, limiting prognostic implications.

**Figure 4:**
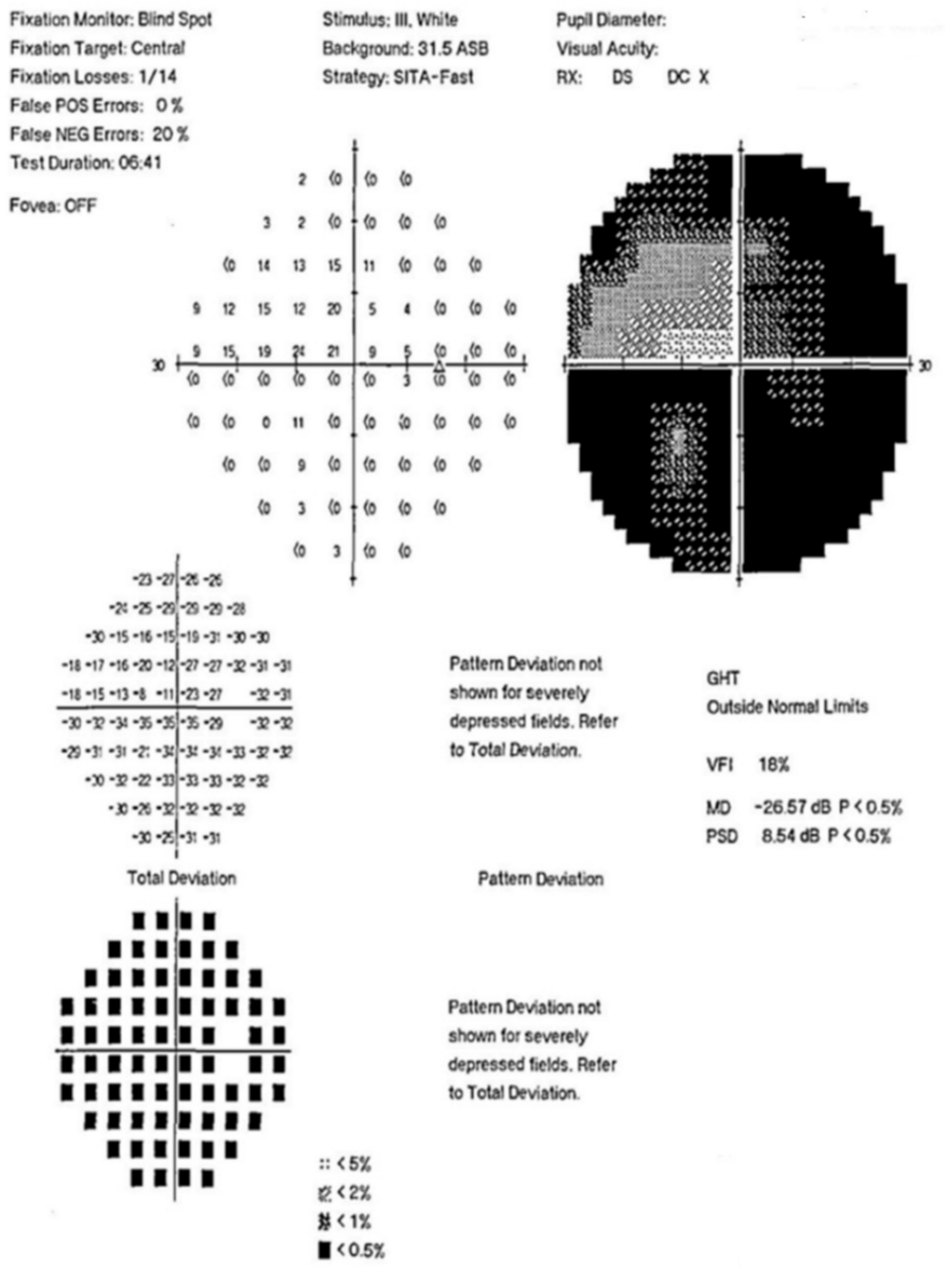
Preoperative visual field examination chart

**Figure 5:**
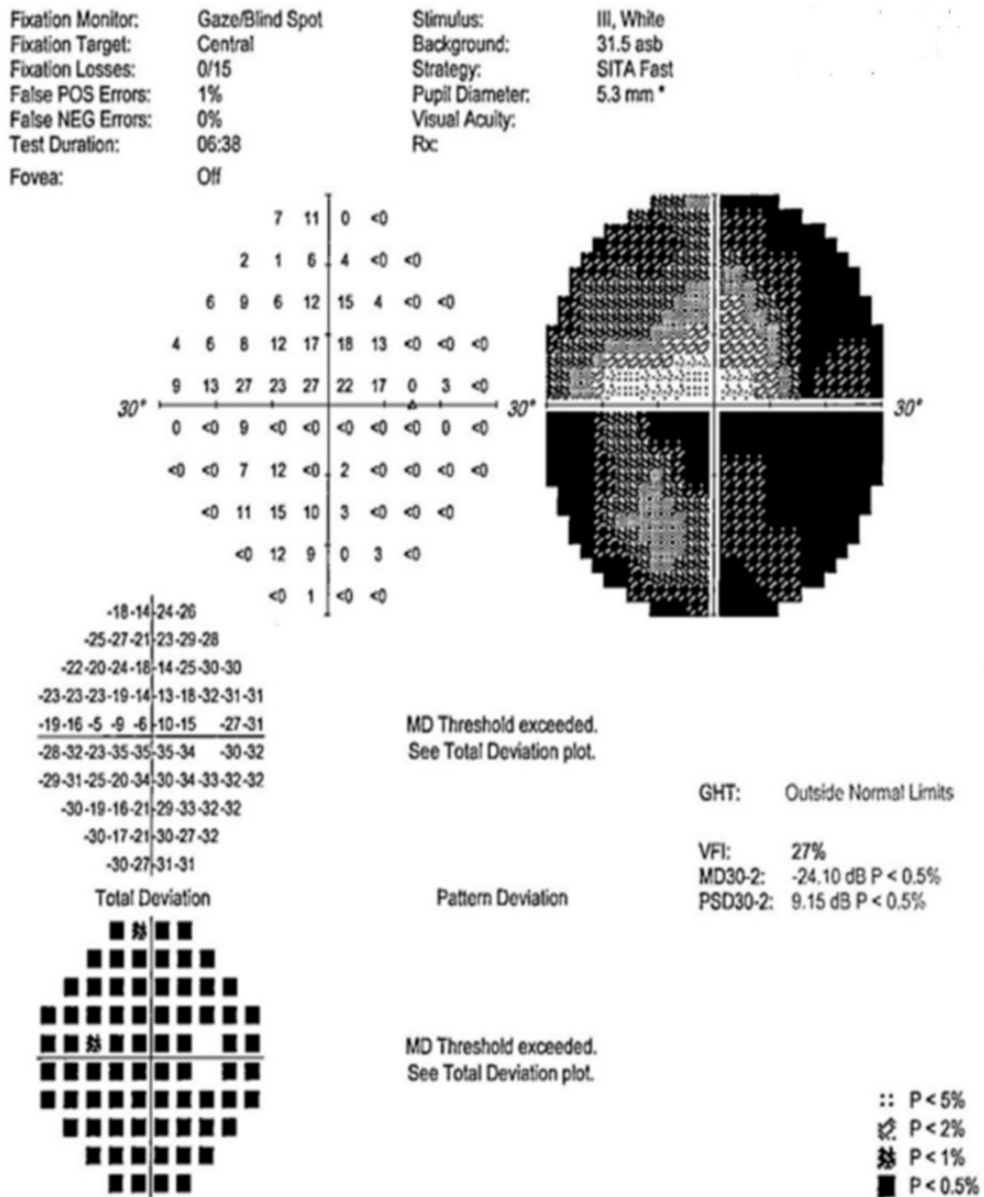
Visual field test chart after the initial fractionated treatment session with the ZAP-X system

**Figure 6:**
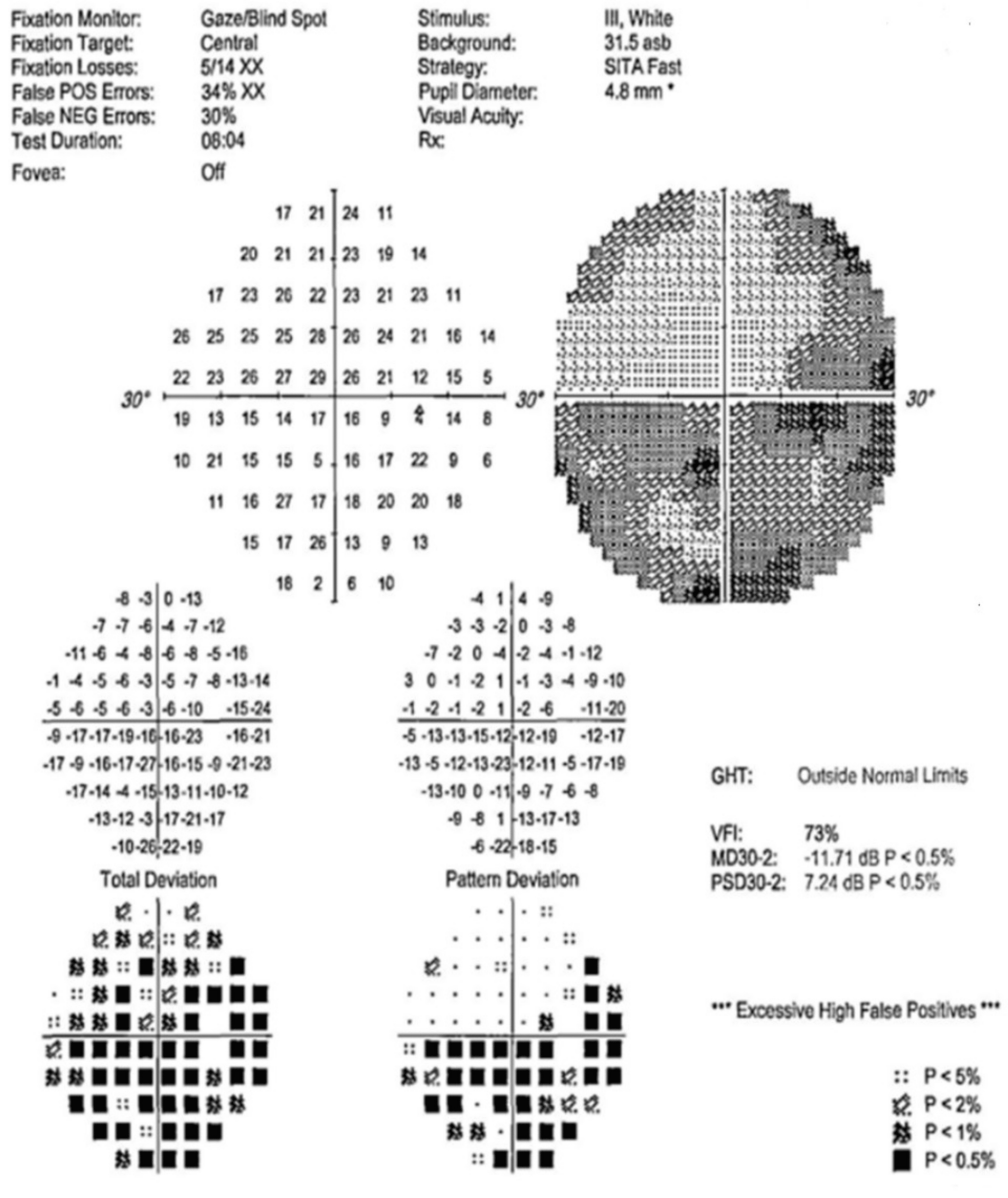
Visual field examination chart following the third fractionated treatment session with the ZAP-X system.

**Figure 7:**
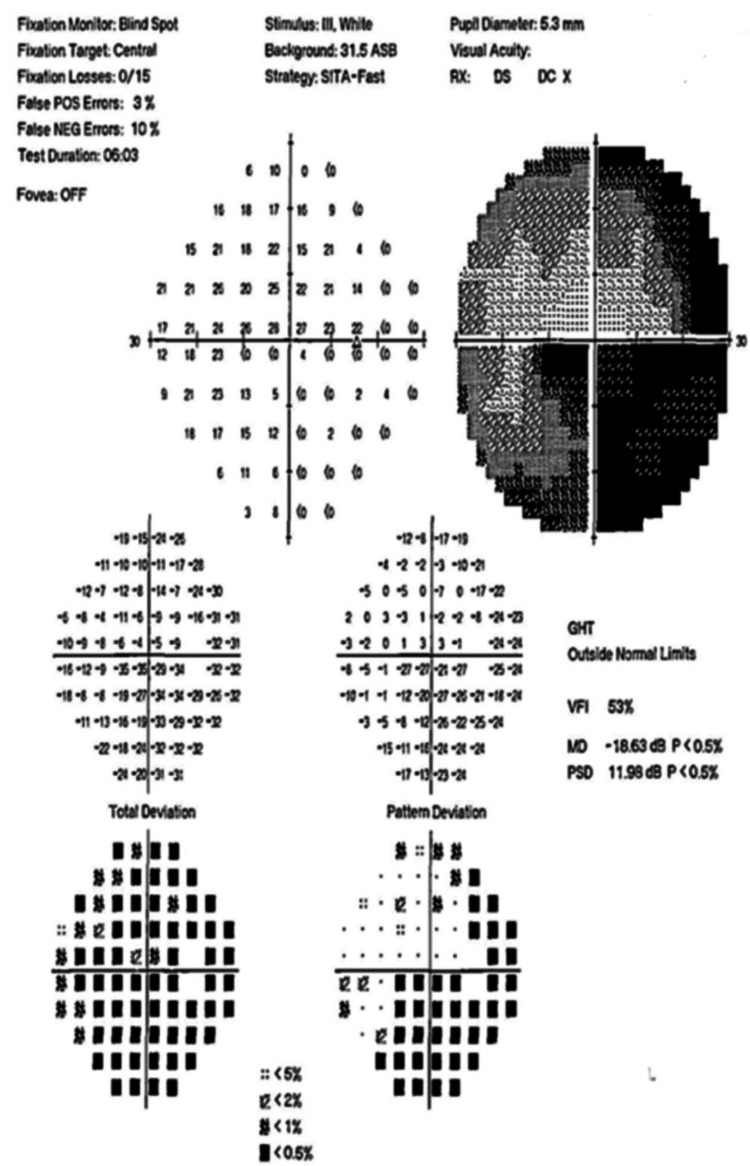
Visual field examination chart one week after ZAP-X treatment

**Figure 8:**
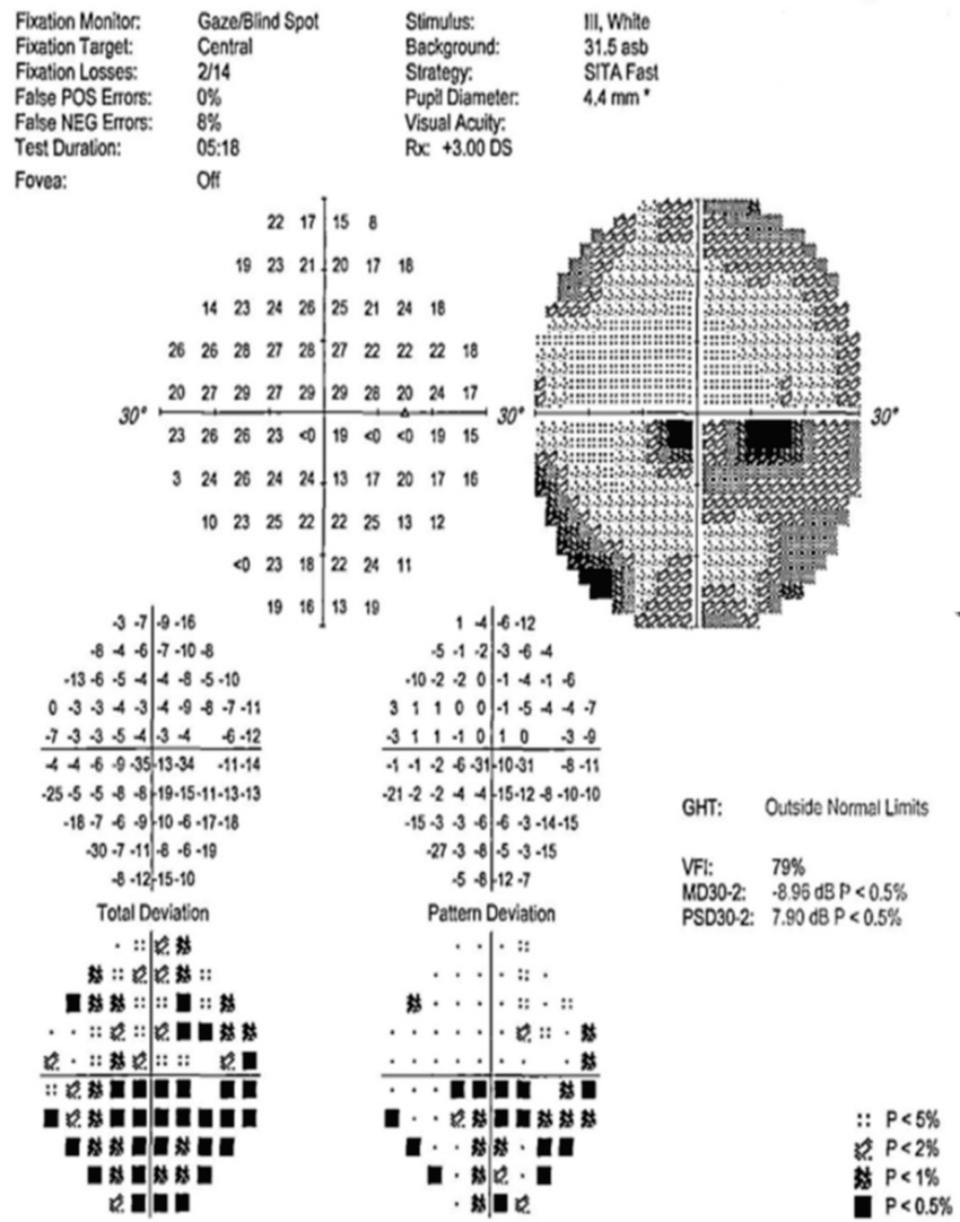
Visual field examination chart one month after ZAP-X treatment

**Figure 9:**
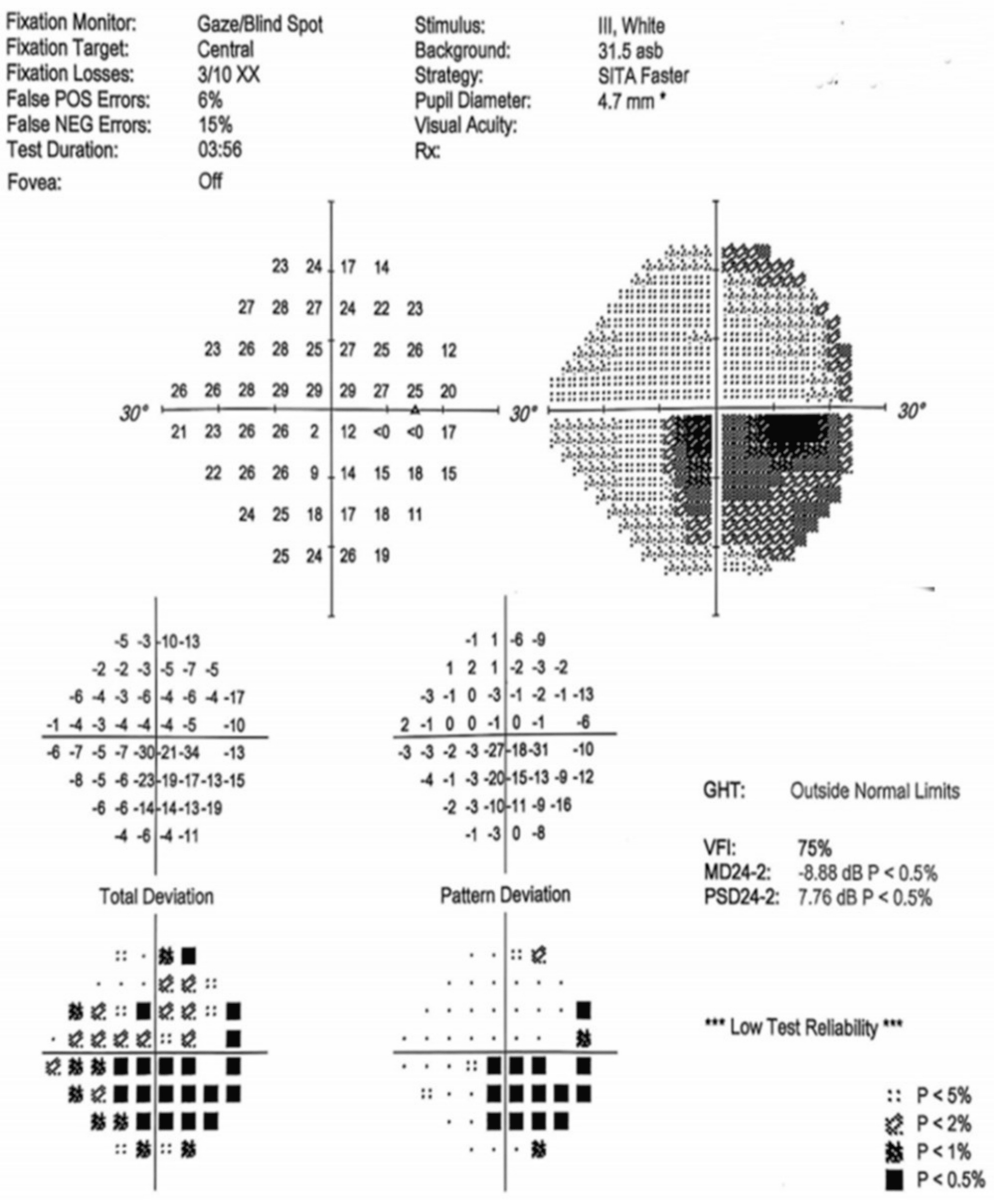
Visual field examination chart three months after ZAP-X treatment

**Figure 10:**
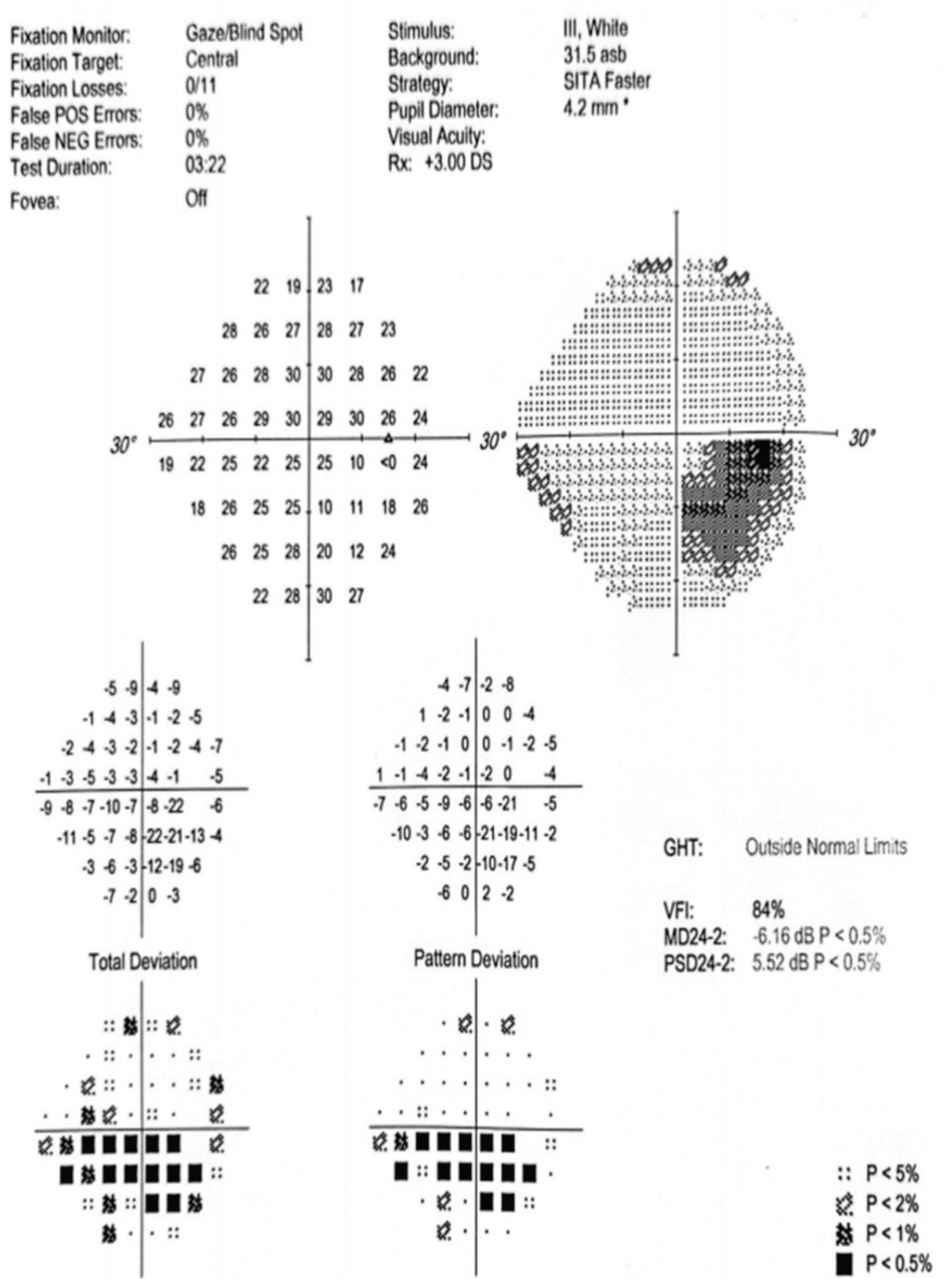
Visual field examination chart 18 months after ZAP-X treatment

Figures 4-10 present this case involving a patient with ONSM who underwent the first fractionated treatment using ZAP-X SRS. Following the initial treatment, the visual field index (MD) improved by 9% (from 18% to 27%; Figure 5), and the mean sensitivity of the visual field increased by 2.4 dB (from -26.57 to -24.10). After the third fractionated treatment, the MD improved by 55% (from 18% to 73%; Figure 6), and the mean sensitivity improved by 14.8 dB (from -26.57 to -11.71). One week after treatment, there was a brief regression in therapeutic effect (Figure 7), but at the one-month follow-up, the MD had improved by 61% (from 18% to 79%, Figure 8), and the mean sensitivity had increased by 16.6 dB (from -26.57 to -8.95). The patient’s corrected visual acuity improved from 0.1 to 0.8 and remained stable across multiple follow-ups. At the 18-month postoperative review, the MD was 84% (Figure 10), and the mean sensitivity was -6.16 dB.

**Figure 11:**
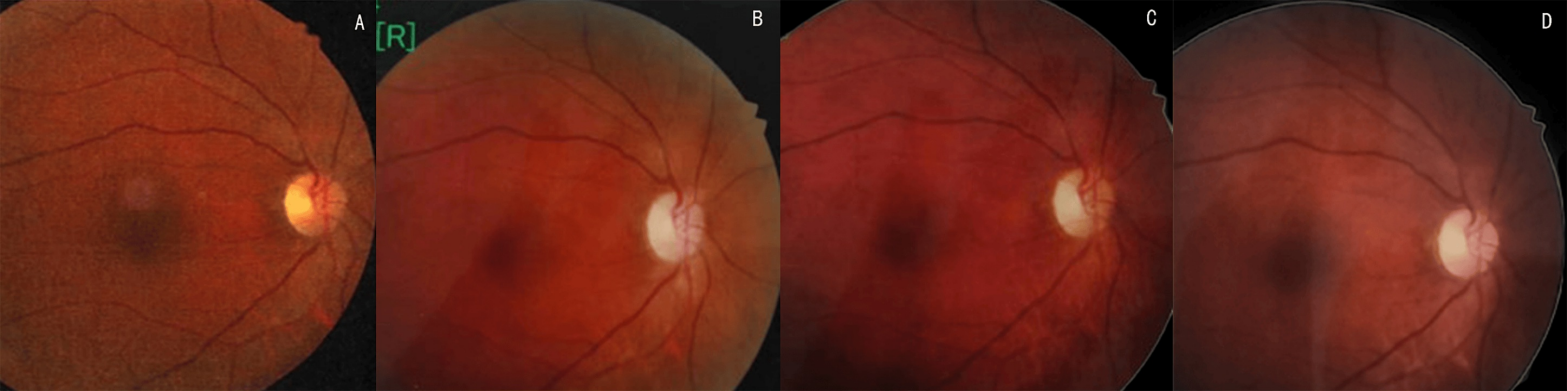
Fundus examination image (A) Preoperative fundus examination image. (B) Fundus examination diagram one week after ZAP-X treatment. (C) Fundus examination diagram three months after ZAP-X treatment. (D) Fundus examination diagram 18 months after ZAP-X treatment

Figure 11 presents a comparison of fundoscopic examinations 18 months postoperatively versus preoperatively, revealing smoother retinal vascular pathways, reducing tortuous shunt vessels, significant alleviation of optic disc edema, and gradual restoration of physiological structures.

## Discussion

The diagnosis and treatment of ONSM are highly challenging. This case highlights the core advantages of the MDT treatment model in ONSM management. Through precise design of personalized treatment timing, the progression of the tumor was controlled while maximizing the preservation of visual function. Additionally, the risk of optic nerve-related complications was significantly reduced. The MDT model demonstrated interdisciplinary decision-making advantages, providing an optimal treatment pathway for cranial tumors.

In this case, the patient underwent a standardized perioperative visual function dynamic monitoring process, comparing multiple pre- and posttreatment visual function assessments. The results demonstrated the safety and efficacy of the Zap-X system in restoring visual function in patients with ONSM. This mechanism may be associated with the advanced technology and dose optimization strategies unique to Zap-X. The treatment of ONSM aims to effectively control the tumor while protecting the optic nerve, which has already been compressed and damaged by the tumor, seeking a more precise balance.

First, the non-coplanar rotational focusing technology and real-time dynamic tracking of the ZAP-X system achieve submillimeter precision (error <0.4 mm), significantly reducing radiation exposure to adjacent optic nerves and the optic chiasm [8]. In this case, the treatment plan referenced the radiation therapy threshold for ONSM (optic nerve dose ≤10 Gy) and adopted a fractionated low-dose regimen (total dose of 23 Gy/5 fractions) [9], thereby reducing the risk of radiation-induced optic neuropathy [3]. Compared to the traditional SRS treatment plan with a 12% incidence of optic nerve complications [10], the automatic target displacement correction of ZAP-X contributed positively to the patient’s visual improvement. As of the completion of this report, the patient had not reported any adverse effects related to visual function, and visual acuity remained stable, fully reflecting the safety of ZAP-X in this case.

Second, it is conventionally believed that rapid tumor shrinkage correlates with rapid visual improvement. Although consecutive posttreatment MRIs in this case did not show significant tumor volume reduction, the marked improvement in the visual field may be related to the ability of radiotherapy to inhibit the tumor's blood supply shunting [11]. Postoperative fundoscopic examinations confirmed that the high precision of the ZAP-X system in targeting the tumor led to apoptosis of endothelial cells in the surrounding tumor vasculature. Additionally, the 3D conformal index (CI = 1.05) of ZAP-X is superior to conventional intensity-modulated radiation therapy (CI = 1.2-1.5). During treatment target delineation, after marking the radiation area of the tumor, the protective characteristics of the optic nerve can be further delineated. This allows for precise targeting of the tumor while simultaneously reducing the radiation dose to surrounding normal tissues, which may explain the rapid progress in visual recovery and the shortened improvement cycle observed in this patient.

The VIDAR™ eye-tracking system integrated into the ZAP-X platform further significantly enhances treatment safety. The established threshold of 0.3 mm facilitates precise monitoring of ocular motion during the procedure, thereby ensuring accurate tumor ablation while minimizing the risk of optic nerve injury.

Although the therapeutic effects in this case are significant, it is important to note that long-term data (greater than five years) on ZAP-X treatment for ONSM, as well as clinical data on late recurrence and IRON risks, remain limited. Future research should focus on expanding sample sizes and conducting prospective controlled studies to collect more clinical data related to Zap-X treatment, further exploring and validating the system’s efficacy and safety.

This case demonstrates the unique value of the ZAP-X system in precision, safety, and visual function preservation during ONSM treatment. Particularly, the fractionated high-dose model may offer a superior option for visual recovery in optic nerve-related tumors.

## Conclusions

Globally, the clinical treatment model for ONSM has evolved over the past decade from traditional surgical intervention to SRS, providing significant clinical value in preserving patients’ vision and achieving better prognoses. As the latest generation of high-precision SRS equipment, ZAP-X continues to explore its potential in treating various intracranial tumors. The rapid visual recovery observed in this case shortly after treatment is extremely rare, greatly enhancing our understanding of the precision of the ZAP-X system. Based on this, we plan to conduct prospective clinical trials to systematically evaluate the safety and efficacy parameters of this equipment in treating different pathological types of cranial tumors, thereby providing evidence-based support for improving the SRS treatment system.
